# Patterns of Recent National Institutes of Health Funding in Family Medicine: Analysis Using the NIH Research Portfolio Online Reporting Tools Expenditures and Results System

**DOI:** 10.7759/cureus.5847

**Published:** 2019-10-06

**Authors:** Erich J Berg, John Ashurst

**Affiliations:** 1 Medicine, Arizona College of Osteopathic Medicine, Phoenix, USA; 2 Emergency Medicine, Kingman Regional Medical Center, Kingman, USA

**Keywords:** family medicine, national institutes of health (nih), r01, gender, osteopathic

## Abstract

Introduction

Despite a call for increased research by family-medicine physicians, there is no data on the demographics of those awarded a National Institutes of Health (NIH) R01 grant.

Objective

The purpose of the study was to assess recent NIH R01 funding trends over the last decade in family medicine.

Methods

A retrospective analysis of NIH R01 grant funding in family medicine was conducted by extracting demographic data from the NIH’s Research Portfolio Online Reporting Tools Expenditures and Results (RePORTER) database from 2008 through 2017. Demographics were reported as percentages, and comparisons of the groups were performed by the t-test.

Results

From 2008 to 2017, the NIH awarded 139 R01 grants to principal investigators (PI) in family medicine. Males comprised 51.80% of all awardees, and those holding a doctorate of medicine (MD) made up 43.88% of the awardees. No one holding a Doctorate of Osteopathic Medicine (DO) degree received an NIH R01 grant during the timeframe studied. A total of 81.97% of all MDs held a dual degree. When gender and degree were considered, no statistical difference was observed for the total amount of dollars awarded.

Conclusion

For the years studied, a disparity related to medical degrees was noted for those family-medicine physicians who received an NIH R01 grant. However, no gender disparity was observed.

## Introduction

The National Institutes of Health (NIH) is the leading source of funding for biomedical research in the US [1]. The NIH R01 grant is the oldest granting mechanism provided by the NIH. An R01 grant supports a discrete project over three to five years and is typically investigator-initiated or solicited by a request for applications [1]. 

In 2002, the North American Primary Care Research Group Committee on Building Research Capacity and the Academic Family Medicine Organizations Research Subcommittee noted: “all family physicians have a role in the generation and application of new knowledge to improve the health of individuals, families, and communities” [2]. The organizations suggested that the only way to achieve this goal is to increase the number of family-medicine physician-scientists in the community. Increasing the family-medicine research workforce and infrastructure, however, lies within establishing protected academic time and relies on securing grant funding, tantamount to changing the current culture in the field [2].

Research has shown that only a small percentage of NIH grants are awarded to the discipline of family medicine [3]. This may be attributed to the fact only a small percentage of general-practice physicians are engaged in scholarly activity [4]. A previous study has noted that over two-thirds of all grants received in family medicine between 2002 and 2006 were R type (research) grants with over half being distinguished as an R01 grant [5]. However, the principal investigators (PIs) who received a grant in family medicine were more likely to be non-physician researchers [6]. In this study, the authors sought to determine the recent trends in NIH R01 grant funding in family medicine.

## Materials and methods

After gaining approval from the institutional review board, we queried the NIH Research Portfolio Online Reporting Tools Expenditures and Results (RePORTER) search engine (http://projectreporter.nih.gov) by using “family medicine” as a keyword on pages that documented new R01 grants issued between the fiscal years of 2008 and 2017. PIs were categorized by gender (male or female), medical degree (osteopathic or allopathic), and other degrees (Ph.D., DSc, MPH, etc.). Physician-scientists were further categorized into those holding a dual degree (a combination of a medical degree and non-medical degree) or those with only a medical degree. The public biographies of PIs displayed by their affiliated institutions were reviewed to aid in this process. Secondarily, the total dollar amount awarded as a grant to each PI was recorded. PIs were not excluded if any of them had been awarded more than one grant over the timeframe studied, and each grant awarded was tallied. The total R01 grant amount was rounded to the nearest dollar. Comparisons of the proportions of gender and degree(s) from each year were determined by using simple descriptive statistics. Comparisons of each group were completed by the t-test. 

## Results

Over the decade studied, a total of 139 R01 grants were found to be awarded to family-medicine departments, which amounted to a total of $81,466,166. 51.8% (79/139) of these grants was awarded to males and 43.88% (61/139) was awarded to allopathic physician-scientists (MD). No osteopathic physician-scientist (DO) was awarded an R01 grant in family medicine over the timeframe studied. On average, males were awarded larger grants based upon dollar amount as compared to their female counterparts, but no statistical difference was noted ($586,905 vs $585,209; p = 0.49). PIs who held a medical degree were awarded less grant money in terms of total dollar amount as compared to those holding a degree designated as "other" ($570,530 vs $598,254; p = 0.30). A total of 81.97% of MDs who had received an R01 grant over the timeframe studied held a dual degree.

When gender and degree were considered, the degree designated as "other" comprised 70.15% (47/67) of all grants awarded to the female gender (Table 1). Those females with a degree designated as "other" were awarded grants with larger total dollar amounts, but this was not statistically significant ($612,765 vs $520,453; p = 0.20) (Table 2). A total of 90% (18/20) of all female MDs held dual degrees. However, no statistically significant difference was noted in terms of the total dollar amount awarded per grant between female MDs holding a dual degree and those with only a medical degree ($525,616 vs $473,979; p = 0.34) (Table 3).

**Table 1:** Percentage of MDs, DOs, and those holding a degree designated as "other" receiving NIH R01 grants in family medicine from 2008 to 2017 DO: Doctor of Osteopathic Medicine; MD: Doctor of Medicine; NIH: National Institutes of Health

Gender	Degree designation		
	MD	DO	Other
Male	56.94% (41/72)	0% (0/72)	43.06% (31/72)
Female	29.85% (20/67)	0% (0/67)	70.15% (47/67

**Table 2:** Average NIH R01 dollar amount awarded to the different genders and degrees in family medicine from 2008 to 2017 MD: Doctor of Medicine, NIH: National Institutes of Health

Gender	Degree designation		
	MD	Other	P-value
Male	594,958	576,254	0.50
Female	520,453	612,765	0.20

**Table 3:** Average family medicine NIH R01 grants in dollar amount awarded to allopathic physicians with an MD and allopathic physicians holding a dual degree based on gender MD: Doctor of Medicine, NIH: National Institutes of Health

Gender	Degree designation		
	MD	Dual Degree	P-value
Male	687,501	568,930	0.02
Female	473,979	525,616	0.34

When males were considered, 43.15% (31/72) held a degree designated as "other", but MDs were awarded larger grants based on total dollar value ($594,958 vs $576,254; p = 0.50) (Tables 1 and 2). A total of 78.05% of males with an MD degree held dual degrees and a statistically significant difference was noted between MD males with dual degrees and those with only a medical degree when the total dollar amount of grant awarded was considered ($568,930 vs $687,501; p = 0.02) (Table 3).

## Discussion

As the largest primary-care specialty, family medicine accounts for the highest number of medical encounters in the US but receives very few research dollars from the NIH [3,7]. On average, traditional scientists or those holding other degrees received more research dollars as compared to their physician-scientist counterparts. One reason for these findings could be related to the relatively small amount of time family physicians spend conducting research. In a recent study by the American Board of Family Medicine, very few family medicine physicians were found to spend more than 10% of their time conducting research, with more than 95% stating that they do not conduct research in any capacity [4]. 

When gender was considered, both the number of awards and the total dollar amount per award were relatively equal. This is the first report that observed near-equality among the genders for NIH R01 grants in family medicine. This finding could be related to recent trends in reported scholarly activity. Following the landmark article by Jagsi et al, a heightened awareness was placed on the gender disparity in scholarly activity, and numerous other publications have further promoted research on gender disparity in scholarly activity [8]. However, data has also shown that female family physicians spend more time conducting research in family medicine as compared to their male counterparts [4]. These two factors may have crucially influenced the findings in our study. 

This investigation also found that no osteopathic physicians were awarded an R01 grant in family medicine during the timeframe studied. Although this finding appears problematic due to the association between osteopathic physicians and primary-care specialties, it is consistent with a previous analysis of emergency-medicine and general-surgery R01 grants [9,10]. Research participation has historically been under-emphasized at osteopathic institutions, and a paucity of scholarly activity exists within osteopathic medical schools. Between 2006 and 2010, 28 osteopathic medical schools together managed to produce only 1,843 published manuscripts, averaging slightly more than 13 publications per year per school [11]. In 2011, osteopathic medical schools were ranked last in NIH research funding among the 17 various types of educational institutions [11]. It is unclear as to why there is a low number of NIH grants and manuscript publications at osteopathic medical schools, and further research into the scholarly activity at osteopathic medical schools is needed. 

## Conclusions

Based on the current study, we find no significant gender disparity in the awarding of NIH R01 grants. However, when the degrees of those who received the grants were considered, a disparity exists, especially considering that no osteopathic physician was awarded an R01 grant over the time period studied. Moreover, non-physician scientists are generally found to receive more grants than physician-scientists. 
